# Deep sequencing identifies novel and conserved microRNAs in peanuts (*Arachis hypogaea *L.)

**DOI:** 10.1186/1471-2229-10-3

**Published:** 2010-01-05

**Authors:** Chuan-Zhi Zhao, Han Xia, Taylor Price Frazier, Ying-Yin Yao, Yu-Ping Bi, Ai-Qin Li, Meng-Jun Li, Chang-Sheng Li, Bao-Hong Zhang, Xing-Jun Wang

**Affiliations:** 1 High-Tech Research Center, Shandong Academy of Agricultural Sciences; Key Laboratory of Crop Genetic Improvement and Biotechnology, Huanghuaihai, Ministry of Agriculture; Jinan 250100, PR China; 2 Key Laboratory for Genetic Improvement of Crop, Animal and Poultry of Shandong Province, Ji'nan 250100, PR China; 3 Department of Biology, East Carolina University, Greenville, NC 27858, USA; 4 Key Laboratory of Crop Heterosis and Utilization (MOE) and State Key Laboratory for Agrobiotechnology, Beijing Key Laboratory of Crop Genetic Improvement, China Agricultural University, Beijing 100094, PR China; 5 Key Laboratory of Crop Genomics and Genetic Improvement (MOA), Beijing Key Laboratory of Crop Genetic Improvement, China Agricultural University, Beijing 100094, PR China

## Abstract

**Background:**

MicroRNAs (miRNAs) are a new class of small, endogenous RNAs that play a regulatory role in the cell by negatively affecting gene expression at the post-transcriptional level. miRNAs have been shown to control numerous genes involved in various biological and metabolic processes. There have been extensive studies on discovering miRNAs and analyzing their functions in model species, such as *Arabidopsis *and rice. Increasing investigations have been performed on important agricultural crops including soybean, conifers, and *Phaselous vulgaris *but no studies have been reported on discovering peanut miRNAs using a cloning strategy.

**Results:**

In this study, we employed the next generation high through-put Solexa sequencing technology to clone and identify both conserved and species-specific miRNAs in peanuts. Next generation high through-put Solexa sequencing showed that peanuts have a complex small RNA population and the length of small RNAs varied, 24-nt being the predominant length for a majority of the small RNAs. Combining the deep sequencing and bioinformatics, we discovered 14 novel miRNA families as well as 75 conserved miRNAs in peanuts. All 14 novel peanut miRNAs are considered to be species-specific because no homologs have been found in other plant species except ahy-miRn1, which has a homolog in soybean. qRT-PCR analysis demonstrated that both conserved and peanut-specific miRNAs are expressed in peanuts.

**Conclusions:**

This study led to the discovery of 14 novel and 22 conserved miRNA families from peanut. These results show that regulatory miRNAs exist in agronomically important peanuts and may play an important role in peanut growth, development, and response to environmental stress.

## Background

MicroRNAs (miRNAs), initially discovered in *C. elegans *[1], are a large group of small endogenous RNAs [2-4] that widely exist in animals [5], plants [6], and in some viruses [7]. Increasing evidence demonstrates that miRNAs play an important function in many biological and metabolic processes, including tissue identity, developmental timing, and response to environmental stress [8,9]. However, miRNAs do not directly control plant growth and development. In contrast, miRNAs negatively control gene expression by targeting protein coding gene mRNAs for cleavage or repressing protein translation [2,3].

miRNAs are first transcribed from miRNA genes, located mainly in the intergenic genomic region, by RNA polymerase II [10-12]. There are also a small number of miRNA genes located inside the protein coding genes. For these miRNAs, the transcription orientation is the same as the protein coding gene, indicating that they are transcribed together [2,13]. Following transcription and several post-transcriptional modifications using different enzymes (Dicer, Hen1, and other enzymes), long primary miRNA transcripts (pri-RNAs) are processed to generate miRNA precursors (pre-miRNAs) and eventually mature miRNAs [14]. Although the length of mature miRNA sequences varies from 16 to 29 nucleotides with an average of 22-nt, a majority of mature miRNAs are 21 to 23-nt in length [15]. DCL1 is a key enzyme in miRNA biogenesis and mutating this gene results in globally decreased miRNA levels in plants, ultimately resulting in pleiotropic defects [16,17]. In addition, HEN1 and HYL1 also play important roles in miRNA biogenesis in plants; mutating these two genes results in severe defects during various developmental stages of plant growth, including vegetation maturity and proper formation of reproductive organs [18-20].

miRNAs are involved in plant responses to the environment and several miRNAs are up-regulated or down-regulated by abiotic stress, including high salinity, drought, and low temperatures [21,22]. Furthermore, the targets of several miRNAs are genes that play important roles in stress tolerance, including the gene encoding Cu/Zn SOD [23-25]. miR393 targets auxin receptor genes, such as TIR1, AFB2, and AFB3, which lower auxin signals and inhibit the pathogen *P. syringae *[26]. miRNAs are also induced by pathogens, which suggests miRNAs are involved in plant-microorganism interactions such as symbiosis events with legumes and rhizobia bacteria [27,28]. Increasing evidence demonstrates that miRNAs might provide a novel platform to better understand plant development and resistance to biotic as well as abiotic stresses.

Currently, 9539 mature miRNAs have been discovered and deposited in the public available miRNA database miRBase (Release 13.0, March 2009; http://microrna.sanger.ac.uk/sequences/index.shtml) [29]. These miRNAs include 1763 miRNAs from 24 plant species. Although numerous miRNAs have been identified in plants, a majority of them were obtained from model species such as *Oryza sativa *(377), *Populus trichocarpa *(234), *Physcomitrella patens *(230), *Arabidopsis thaliana *(187), and *Vitis vinifera *(140). This could be attributed to the fact that the entire genomes of these organisms have already been sequenced and are readily available. Even so, few miRNAs have been reported in important agricultural crops. Peanut is widely cultivated and is one of the most important economic and oil crops in China, the USA, and around the world. To date, no miRNA-related research has been performed on peanuts.

There are two major methods used in identifying miRNAs: (1) a direct cloning method, using miRNA-enriched libraries, combined with computational and experimental verification [21,30-32] and (2) computational identification. Several investigations have shown that some miRNAs are highly conserved throughout evolution and can be found in mosses to higher flowering plants [31,33,34] This suggests a powerful strategy for identifying miRNAs using comparative genomics. By performing Blastn searches, using already known miRNAs, against Genbank databases including genome survey sequences (GSS), high through-put genomic sequences (HTGS), expressed sequence tags (ESTs), and non-redundant (NR) nucleotides, hundreds of miRNAs have been identified in plants. Currently, several laboratories have adopted this method in order to identify miRNAs [34-41]. However, this method is limited by the number of nucleotide sequences available in the database. For peanut, the genome has not been completely sequenced and there are only a limited number of peanut ESTs in the database. This does not make computational prediction an effective choice for discovering peanut miRNAs. In this study, we employed the next generation high through-put sequencing technology to sequence and identify peanut miRNAs. Based on our study, we have identified 75 conserved miRNAs as well as 14 novel miRNAs in peanuts. Quantitative real time PCR (qRT-PCR) analysis shows that these miRNAs are expressed in peanuts.

## Results and Discussion

### Peanut has a complex small RNA population

To date, 92,988 peanut ESTs, including 86,724 ESTs from cultivated peanuts and 6,264 ESTs from wild-type peanuts, have been deposited in the NCBI EST database. These sequences are minor compared with the 2,800-Mb genome of the allotetraploid cultivated peanut or even the genome of the diploid wild-type peanut. Previous studies have demonstrated, using computational approaches and EST analysis, that only three conserved miRNAs exist in peanut [34,38,41]. With the limited amount of peanut ESTs in the EST database, it is not possible to perform a comprehensive study of peanut miRNAs using only a computational analysis. Experimental cloning and subsequent functional analysis, combined with computational prediction, appears to be the most effective method to identify peanut miRNAs.

Next generation high through-put sequencing, including 454 and Solexa technologies, provides a powerful tool for miRNA cloning. By using the high through-put Solexa sequencing technology, a total of 6,009,541 sequences were obtained from a small RNA library, which was constructed from the cultivated peanut variety Fenghua-1. After removing the low quality sequences and adapter sequences, 4,994,631 sequences were obtained with 3-30 nt in length, among which 4,598,005 sequences ranged from 18-30 nt in length. After further removing tRNAs (437,117), rRNAs (133,410), snRNAs (1,282), and snoRNAs (240), a total of 4,025,956 small RNA sequences were obtained. Although some small RNAs were very high in abundance and present thousands of times in our dataset, the majority of small RNAs were sequenced only a few times. For example, 2,232,910 out of 4,598,005 small RNAs were sequenced only one time in our experiment. This result suggests that (1) the expression of different small RNAs in peanut varies drastically and (2) the small RNA survey in peanut is far from saturated. This also suggests that peanut contains a large and diverse small RNA population.

**Figure 1:**
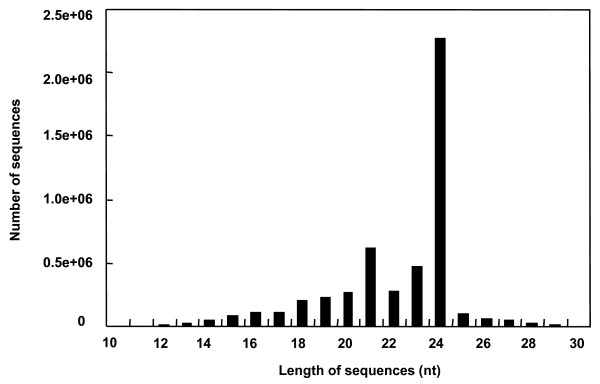
**Length distribution and abundance of the sequences**.

In peanut, the size of the small RNAs was not evenly distributed (Figure 1). Among these sequences, the number of 24-nt sequences was significantly greater than shorter or longer sequences (Figure 1) and accounted for 45% of the total sequence number. This result was consistent with that of Medigcago [42] and rice [43], as well as *Arabidopsis *454 sequencing results [44]. In Arabidopsis, the 24-nt small RNAs accounted for about 60% of its small RNA transcriptome [45]. However, the size distribution differs from wheat and conifer sequences obtained through 454 high through-put sequencing [43,46] and Chinese yew sequences obtained through Solexa sequencing [47]. In conifer, the fraction of 24-nt RNAs was very small (2.6%) due to the lack of DCL3, the enzyme that matures 24-nt RNAs in angiosperms [43,48]. In total, 620,060 sequences (13.5%) with 21-nt, which is the typical length of plant mature miRNAs, represented the second highest abundance of sequences in the peanut library.

### Identification of conserved peanut miRNAs

To identify conserved miRNAs in peanuts, all small RNA sequences were Blastn searched against the currently known miRNAs in the miRNA database miRBase (March 9, 2009). In total, 1,763 known miRNAs from diverse plant species were utilized in order to identify conserved peanut miRNAs from the small RNA dataset.

After Blastn searches and further sequence analysis, a total of 75 conserved miRNAs were identified in peanuts and these miRNAs belong to 22 miRNA families (Table 1). Of the 22 miRNA families, three miRNA families (miR156/157, miR166, and miR167) were predicted [34,38,41] using a comparative genomics-based strategy [38]. The identified miRNA families have been shown to be conserved in a variety of plant species. For example, miR156/157, miR159/319, miR166, miR169, and miR394 have been found in 51, 45, 41, 40, and 40 plant species, respectively [34,38,41]. In this study, we also tried to identify the precursor sequences for the 75 conserved peanut miRNAs. However, due to the fact that the peanut genome has not been fully sequenced, the pre-miRNAs and their secondary structures were only identified for nine miRNAs (Additional file 1).

**Table 1:** Conserved miRNAs from peanut

miRNA family	Name	Sequence(5'-3')	Length (nt)	Reference miRNA	Conserved in other plants				Reads
						
					ath	ptc	vvi	osa	
156/157	ahy-MIR156a	ugacagaagagagugagcac	20	ath-miR156a	++	++	++	++	17058
	ahy-MIR156b	ugacagaagagagugagcaca	21	bna-miR156a	+	+	+	+	255
	ahy-MIR156c	cugacagaagauagagagcac	21	smo-miR156b	+	+	+	+	43
	ahy-MIR156e	ugacagaggagagugagcac	20	vvi-miR156e	+	+	++	+	8
	ahy-MIR156 g	cgacagaagagagugagcac	20	ath-miR156 g	++	+	+	+	15
	ahy-MIR156 h	ugacagaagaaagagagcac	20	ath-miR156 h	++	+	+	+	4
	ahy-MIR156k	ugacagaagagagggagcac	20	ptc-miR156k	+	++	++	+	69
	ahy-MIR156f	uugacagaagaaagagagcac	21	smo-MIR156c	+	+	+	+	4
	ahy-MIR157a	uugacagaagauagagagcac	21	ath-miR157a	++	++	++	+	95381
	ahy-MIR157d	ugacagaagauagagagcac	20	ath-miR157d	++	+	++	+	3967
	ahy-MIR157k	ugacagaagagagcgagcac	20	zma-miR156k	+	+	+	+	67

159	ahy-MIR159a	uuuggauugaagggagcucua	21	ath-miR159a	++	++	++	+	66
	ahy-MIR159b	uuuggauugaagggagcucuu	21	ath-miR159b	++	+	+	+	41
	ahy-MIR319a	uuggacugaagggagcucccu	21	ath-miR319a	++	+	+	+	12
	ahy-MIR319b	uuggacugaagggagcuccc	20	mtr-miR319	+	++	+	+	5

160	ahy-MIR160a	ugccuggcucccuguaugcca	21	ath-miR160a	++	++	++	++	41
	ahy-MIR160b	ugccuggcucccugaaugcca	21	osa-miR160f	+	++	++	++	4

162	ahy-MIR162a	ucgauaaaccucugcauccag	21	ath-miR162a	++	++	++	++	94

164	ahy-MIR164a	uggagaagcagggcacgugca	21	ath-miR164a	++	++	++	++	4116
	ahy-MIR164d	uggagaagcagggcacgugcu	21	osa-miR164d	+	+	+	++	88
	ahy-MIR164c	uggagaagcagggcacgugcg	21	ath-miR164c	++	+	+	+	4
	ahy-MIR164d	uggagaagcaggguacgugca	21	osa-miR164c	+	+	+	++	1

166	ahy-MIR165a	ucggaccaggcuucauccccc	21	ath-miR165a	++	+	+	+	40
	ahy-MIR166a	ucggaccaggcuucauucccc	21	ath-miR166a	++	++	++	++	9577
	ahy-MIR166d	ucggaccaggcuucauuccccu	22	vvi-miR166d	+	+	++	+	9
	ahy-MIR166 g	ucggaccaggcuucauuccuc	21	osa-miR166 g	+	+	++	++	3647
	ahy-MIR166 h	ucggaccaggcuucauuccc	20	zma-miR166 h	+	+	+	+	8585
	ahy-MIR166j	ucggaucaggcuucauuccuc	21	osa-miR166j	+	+	+	++	8
	ahy-MIR166 m	ucggaccaggcuucauucccu	21	osa-miR166 m	+	+	+	++	35
	ahy-MIR166n	ucggaccaggcuucauuccuu	21	ptc-miR166n	+	++	+	+	13
	ahy-MIR166e	ucgaaccaggcuucauucccc	21	osa-MIR166e	+	+	+	++	3
	ahy-MIR166k	ucggaccaggcuucaaucccu	21	osa-miR166k	+	+	+	++	1
	ahy-MIR166b	ucggaccaggcuucauuccccc	22	vvi-miR166c	+	+	++	+	5

167	ahy-MIR167a	ugaagcugccagcaugaucua	21	ath-miR167a	++	++	++	++	2572
	ahy-MIR167b	ugaagcugccagcaugaucuaa	22	bna-miR167a	+	+	+	+	34
	ahy-MIR167c	ugaagcugccagcaugaucuc	21	vvi-miR167c	+	+	++	+	15
	ahy-MIR167d	ugaagcugccagcaugaucugg	22	ath-miR167d	+	+	+	+	224
	ahy-MIR167e	ugaagcugccagcaugaucug	21	osa-miR167d	+	++	++	+	34
	ahy-MIR167f	ugaagcugccagcaugaucuu	21	ptc-miR167f	+	++	+	+	8767

168	ahy-MIR168a	ucgcuuggugcaggucgggaa	21	ath-miR168a	++	++	++	+	19898
	ahy-MIR168b	ucgcuuggugcagaucgggac	21	osa-miR168a	+	+	+	++	86

169	ahy-MIR169b	cagccaaggaugacuugccgg	21	ath-miR169b	++	++	++	++	66
	ahy-MIR169e	uagccaaggaugacuugccgg	21	osa-miR169e	+	+	+	++	1
	ahy-MIR169a	cagccaaggaugacuugccga	21	ath-miR169a	++	++	++	++	1
	ahy-MIR169 m	gagccaaggaugacuugccgg	21	vvi-miR169 m	+	+	++	+	1

171	ahy-MIR171b	ugauugagccgugccaauauc	21	osa-miR171b	+	++	+	++	26
	ahy-MIR171c	agauugagccgcgccaauauc	21	ptc-miR171c	+	++	+	+	1
	ahy-MIR171d	ugauugagccgcgucaauauc	21	vvi-miR171b	+	+	++	+	5
	ahy-MIR171f	uugagccgcgccaauaucacu	21	vvi-miR171f	+	+	++	+	3
	ahy-MIR171e	uugagccgugccaauaucac	20	zma-miR171b	+	+	+	+	1
	ahy-MIR171a	uugagccgugccaauaucaca	21	zma-miR171f	+	+	+	+	4

172	ahy-MIR172a	agaaucuugaugaugcugcau	21	ath-miR172a	++	++	++	++	2176
	ahy-MIR172b	agaaucuugaugaugcugca	20	zma-miR172a	+	+	+	+	81
	ahy-MIR172c	agaaucuugaugaugcugcag	21	ath-miR172c	++	+	+	+	58
	ahy-MIR172e	ggaaucuugaugaugcugcau	21	ath-miR172e	++	++	+	++	2

390	ahy-MIR390a	aagcucaggagggauagcgcc	21	ath-miR390a	++	++	++	++	149

393	ahy-MIR393a	uccaaagggaucgcauugaucc	22	ath-miR393a	++	+	++	+	2
	ahy-MIR393b	uccaaagggaucgcauugauc	21	osa-miR393	+	++	+	++	6
	ahy-MIR393c	uccaaagggaucgcauugaucu	22	osa-miR393b	+	+	+	++	1

394	ahy-MIR394a	uuggcauucuguccaccucc	20	ath-miR394a	++	++	++	++	8

396	ahy-MIR396a	uuccacagcuuucuugaacug	21	ath-miR396a	++	++	++	++	221
	ahy-MIR396b	uuccacagcuuucuugaacuu	21	ath-miR396b	++	++	+	++	35
	ahy-MIR396d	uccacaggcuuucuugaacug	21	osa-miR396d	+	+	+	++	1
	ahy-MIR396c	uuccacagcuuucuugaacua	21	vvi-miR396a	+	+	++	+	5
	ahy-MIR396e	uuccacagcuuucuugaacu	20	vvi-miR396b	+	+	++	+	2

397	ahy-MIR397a	ucauugagugcagcguugaug	21	ath-miR397a	++	++	++	++	344
	ahy-MIR397c	ucauugagugcagcguugaugu	22	bna-miR397a	+	+	+	+	5
	ahy-MIR397b	uuauugagugcagcguugaug	21	osa-miR397b	+	+	+	++	1

398	ahy-MIR398b	uguguucucaggucgccccug	21	osa-miR398b	+	++	++	++	12

399	ahy-MIR399e	ugccaaaggagauuugcccag	21	osa-miR399e	+	+	+	++	1

408	ahy-MIR408a	augcacugccucuucccuggc	21	ath-miR408	++	++	++	+	105
	ahy-MIR408b	ugcacugccucuucccuggcu	21	ppt-miR408b	+	+	+	+	5

528	ahy-MIR528	uggaaggggcaugcagaggag	21	osa-miR528				++	3

535	ahy-MIR535	ugacaacgagagagagcacgc	21	ppt-miR535a			+	+	1

894	ahy-MIR894	cguuucacgucggguucacc	20	ppt-miR894					2

The abbreviations represent: ath, Arabidopsis thaliana; ptc, *Populus trichocarpa*; vvi, *Vitis vinifera*; osa, *Oryza sativa*. The plus symbols indicate: ++, miRNA sequences of peanut were exactly identical to those in other species; +, miRNA sequences of peanut were conserved in other species but have variations in some nucleotide positions.

**Figure 2:**
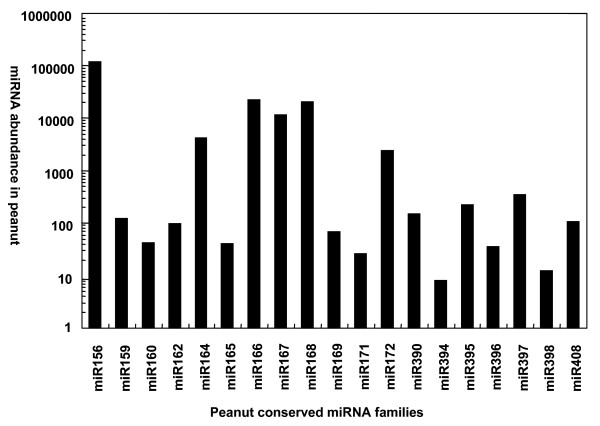
**Abundance of peanut-conserved miRNA families**.

Next generation high through-put sequencing provides an alternative way to estimate expression profiles of protein coding genes and/or miRNA genes [44,46]. Millions of peanut small RNA sequences, generated from Solexa sequencing, allowed us to determine the abundance of various miRNA families and even distinguish between different members of a given family. Interestingly, peanut miRNA families displayed significantly varied abundance from each other. For example, ahy-miR157a, ahy-miR168a, and ahy-miR156a were detected 95,381, 19,898, and 17,058 times respectively (Table 1). In comparison to other plant species, tae-miR169b in wheat and osa-miR169 in rice were the most frequently sequenced miRNAs while miR156 in rice and wheat exhibited low abundance [46]. This suggests a species-specific expression profile for miRNAs. miR156a was also found to be highly expressed in another legume species, Medicago [49]. In *Arabidopsis*, miR156a, located on chromosome 2 [49], targets 10 mRNAs that code for the squamosa promoter-binding protein (SBP) box, which is involved in leaf morphogenesis [50]. Similar to miR156a, miR157a, which is located on chromosome 1 in *Arabidopsis thaliana*, was thought to target mRNAs coding for proteins comprising the SBP box [49]. However, the mechanisms, causing the differential expression profile of a same miRNA in different plant species, are unknown. A majority of peanut miRNAs were only sequenced less than 1,000 times, and some rare miRNAs were detected less than 10 times. Compare with the most abundant miRNA ahy-miR157a, their expression level is about 9,500 times lower (Table 1). miRNAs of moderate abundance included ahy-miR157d, ahy-miR164a, ahy-miR166a, ahy-miR166 g, ahy-miR166a, ahy-miR167f, and ahy-miR172a were detected 2,000-10,000 times in the library. The relative abundance of the 22 conserved peanut miRNA families is represented in Figure 2.

Next generation high through-put sequencing technology also provides a method for distinguishing and measuring miRNA sequences with only a few nucleotide changes. Based on the results from the Solexa sequencing, different family members displayed drastically different expression levels. For example, the abundance of miR156 family varied from 4 read (ahy-miR156f) to 17,058 reads (ahy-miR156a) in the deep sequencing. This was also the case for some other miRNA families, such as ahy-miR164 (from 1 read to 4,116 reads) and ahy-miR166 (from 1 read to 9577 reads). The existence of a dominant member in a miRNA family may suggest that the regulatory role of this family was performed by the dominant member at the developmental time when the samples were collected for RNA extraction. Abundance comparisons of different members in one miRNA family, during various growth conditions or specific developmental stages, may provide valuable information on the role that miRNAs play in plant growth. Expression levels of two members of the ahy-miR159 family (ahy-miR159a and ahy-miR159b) were similar and were detected 66 and 41 times, respectively (Table 1).

### Identification of novel peanut miRNAs

In addition to the identification of conserved miRNAs, 14 novel peanut miRNA families were also identified (Table 2). Only one member was identified in each species-specific miRNA family and the read number for each novel miRNA was much lower than that for the conserved miRNAs. This is consistent with previous conclusions indicating that non-conserved miRNAs are usually expressed at lower levels and with a tissue- or developmental-specific pattern. Therefore, miRNAs identified in this study might represent only a small portion of novel miRNA families found in peanut due to the fact that the small RNA library was constructed from young peanut seedlings grown under normal conditions. Precursors of these novel miRNAs were identified and formed proper secondary hairpin structures, with free energies ranging from -26.91 kcal mol^-1 ^to -132 kcal mol^-1 ^(average of -52.54 kcal mol^-1^) (Table 2, Additional file 1). More importantly, the identification of an anti-sense miRNA (miRNA*) from five novel miRNA candidates provided more evidence to consider them as novel miRNAs. To investigate the conservation of these 14 novel miRNAs in a wide range of plant species, we used these 14 miRNAs as query sequences to perform Blastn searches against all nucleotide sequences in NCBI databases. No homologs were found in any plant species except miRn1, which has a homolog in the soybean EST CD39249. This suggests that these newly identified miRNAs are all peanut-specific miRNAs except miRn1.

**Table 2:** Novel miRNAs identified from peanut

Name	Count	miRNA sequence	Folding energy
ahy-miRn1	656	UAGAGGGUCCCCAUGUUCUCA	-65.9
ahy-miRn2	40	UCACCGUUAAUACAGAAUCCUU	-70.57
ahy-miRn2*	3	AGGAUUCUGUAUUAACGGUGA	-70.57
ahy-miRn3	15	AAUGUAGAAAAUGAACGGUAU	-64.6
ahy-miRn4	12	UGCUGGGUGAUAUUGACAGAAG	-48.72
ahy-miRn5	7	CUGACCACUGUGAUCCCGGAA	-39.5
ahy-miRn6	6	UGACCUUUGGGGAUAUUCGUG	-61.9
ahy-miRn7	5	UCAAUCAAUGACAGCAUUUCA	-39.42
ahy-miRn8	4	UGGUGAUGGUGAAUAUCUUAUC	-38.1
ahy-miRn8*	1	AAGGGAGACGUUUGAAUUAUC	-38.1
ahy-miRn9	3	UGGUGAGUCGUAUACAUACUG	-30.91
ahy-miRn10	3	AUACUUGAGAGCCGUUAGAUGA	-52.8
ahy-miRn10*	1	AUCUAACGACUCUCAGAUAUAAU	-52.8
ahy-miRn11	3	UUAUACCAUCUUGCGAGACUGA	-49.7
ahy-miRn12	4	UGUUACUAUGGCAUCUGGUAA	-40.2
ahy-miRn12*	1	GCCAGGGCCAUGAAUGCAGAU	40.2
ahy-miRn13	3	CGCAAAUGAUGACAAAUAGA	-26.91
ahy-miRn14	11	UUAAUUUCUGAGUUUGUCAUC	-32.57
ahy-miRn14*	1	UUGAUAAGAUAGAAAUUGUAU	-32.57

Besides these 14 identified novel candidate miRNAs, we also discovered two small RNAs, with 701 and 159 reads in our small RNA dataset, which correspond to *Phaseolus vugaris *legume-specific miRS1 and miR2118. These two miRNAs were able to detected in peanut by northern blot analysis [51]. Interestingly, the expression of miR2118 has previously been shown to be induced in *Phaseolus vugaris *by abiotic stress, especially drought and ABA treatment [51]. We did not include these two sequences in the list of novel peanut miRNAs because we could not find their precursor sequences in the current databases. In addition to miRS1 and miR2118, we also found the third small RNA with 137 reads in our dataset that had only one mismatch with *Phaseolus vugaris *miR159.2. A fourth 21-nt small RNA with 729 reads was also identified in our dataset, which had 4 mismatches and one nucleotide missing to compare with *Phaseolus vugaris *miR482*.

Based on the number of detection times and sequences in the small RNA library, novel peanut miRNAs displayed lower expression levels compared to the majority of conserved families. The low abundance of novel miRNAs might suggest a specific role for these miRNAs under various growth conditions, in specific tissues, or during developmental stages. The library enriched only small RNAs that play a role during early seedling stages under normal growth conditions. Whether these low-abundant miRNAs are expressed at higher levels in other tissues and organs, such as flowers, gynophores, pods, or seeds, or whether they are regulated by biotic or abiotic stress, remains to be investigated. Future experiments would provide more insight into the function of these miRNAs.

### Validation of peanut miRNAs

**Figure 3:**
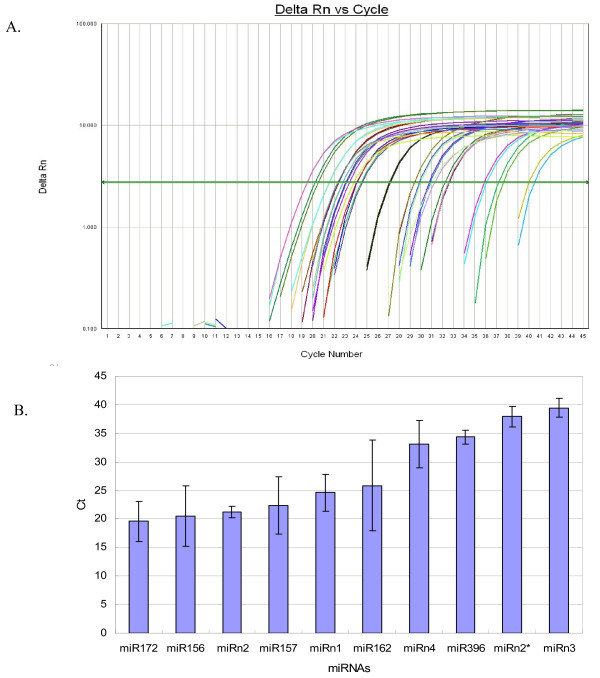
**qRT-PCR validation of the identified peanut miRNAs using high through-put sequencing technology**. A. Amplification plot; B. Threshold cycle. Error bars indicate one standard deviation of three different biological replicates (n = 3).

Stem-loop qRT-PCR is a reliable method for detecting and measuring the expression levels of miRNAs. The stem-loop primers increase the sensitivity of the reactions such that this method can significantly distinguish two miRNAs with only one single nucleotide change [52]. In this study, we adopted this technique to validate and measure the expression of 4 novel miRNAs (miRn1, miRn2 and miRn2*, miRn3, and miRn4) as well as 5 conserved miRNAs (miR156, miR157, miR162, miR172, and miR396). All of these miRNAs were identified in peanut by Solexa sequencing. The qRT-PCR results demonstrate that all tested miRNAs, and one miRNA*, are expressed in peanut leaves (Figure 3). However, the expression levels of the different miRNAs varied.

The results of the qRT-PCR reaction show that conserved miRNAs are expressed in peanut. Based on the threshold cycle (C_T_), miR172 and miR156 were highly expressed with C_T _values of 19.6 ± 3.5 and 20.5 ± 5.3, respectively. In one of our previous studies, we also found that miR172 is highly expressed in cotton leaves [53]. Other studies have shown that conserved miR172 and miR156 play very important roles in plant growth and development [41]. miR156 is involved in *Arabidopsis *leaf development by negatively regulating the Squamosa-promoter binding protein (SBP) [38,42]. miR172 controls flower development by regulating the expression of *apetala2 *(ap2) in *Arabidopsis *[4,43] and glossy 15 in maize [44]. Aberrant expression of miR156 and miR172 in plants disrupts normal leaf and flower development. Compared with miR156 and miR172, the expression levels of miR157 and miR162 are moderate while the expression of miR396 is low. The expression patterns of these miRNAs appear to be related to their function.

Four novel miRNAs and one miRNA*, all identified by Solexa sequencing, were validated by qRT-PCR. The expression levels of the miRNAs differed from one another in peanut leaves. miRn2 and miRn1 were expressed much higher than other tested peanut-specific miRNAs with a C_T _value of 21.2 ± 1.0 and 24.6 ± 3.2, respectively. The expression levels are much lower for miRn3 and miRn2* with C_T _values of 37.9 ± 1.8 and 33.1 ± 4.2. However, more studies need to be performed to elucidate the function that these miRNAS have on the growth and development of peanut.

### Target prediction of peanut miRNAs

To better understand the functions of the newly identified species-specific as well as conserved peanut miRNAs, putative targets of these miRNAs were predicted using the described criteria and methods. The target genes of thirteen conserved and seven novel peanut miRNA families were predicted. Transcription factors, including GRAS family transcription factor, nuclear transcription factor Y subunit and NAC1 were predicted to be potential targets of peanut miRNAs. Furthermore, genes directly involved in protein synthesis, *e.g.*, ribosomal protein S12, were targets of peanut miRNAs. A previous study indicates that auxin signaling is regulated by miRNAs [18]; our current result is consistent with this study and the auxin signaling F-box 3 is a potential target of peanut miR393. Resveratrol synthase, NAM (no apical meristem)-like protein, growth regulator factor 5, basic blue copper protein, endonuclease, a protein kinase, transport inhibitor response 1 and a disease resistance response protein were also predicted to be potential targets of identified peanut miRNAs (Additional file 2).

## Conclusion

For the first time we discovered, through high through-put Solexa sequencing, 14 novel miRNA families and 75 conserved miRNAs, belonging to 22 families, in peanut. Of these 14 novel peanut miRNAs, 13 are peanut-specific because no homologs have been found in other plant species. qRT-PCR analysis demonstrated that both conserved and peanut-specific miRNAs are expressed in peanuts.

## Methods

### Plant materials

Peanuts (*Arachis hypogaea L*. cv. Fenghua-1) were grown in a growth chamber, with a light intensity of 3000 lx, at a relative humidity of 75%, and 26/20°C day/night temperatures. Leaves, stems, and roots from 14-day-old seedlings were collected and immediately stored in liquid nitrogen until total RNA extraction.

### RNA extraction and miRNA cloning

Total RNA was isolated from leaves and roots using Trizol agent (TaKaRa, Dalian, China), according to the manufacturer's instructions. Total RNA was isolated from stems using a modified CTAB method with isopropanol instead of lithium chloride for RNA precipitation [54]. Briefly, one gram of stem tissue was ground to a fine powder using liquid nitrogen and mixed thoroughly with 5 ml of pre-warmed (65°C) extraction buffer (2% CTAB, 2% PVP, 0.1 M Tris-HCl, 2.0 M NaCl, 25 mM EDTA, 2% beta-mercaptoethanol, pH 8.0). The mixture was incubated at 65°C for 5 min and shaken three individual times during the incubation period. After a brief cooling of the mixture, 2.5 ml of chloroform and 2.5 ml of isopropanol were added. The mixture was vortexed for 1 min and then centrifuged at 12000 rpm for 15 min at 4°C. After DNase treatment of the extract, RNA was precipitated at room temperature (25°C) for 10 min using an equal volume of isopropanol. The RNA was resuspended in an equal volume of phenol:chloroform:isopropanol (25:24:1), and then resuspended again with an equal volume of chloroform:isopropanol (24:1). A total of 1/10 volume of 3 M NaOAC (pH 5.2) and 2.5 volumes of cold ethanol were added to precipitate the RNA overnight at -20°C.

To identify as many tissue- or developmental-specific miRNAs as possible, we pooled the total RNAs from leaf, stem, and root samples in an equal fraction ratio. miRNA cloning was performed as described previously by Sunkar and Zhu [21]. Briefly, 0.5 M NaCl and 10% PEG8000 were used to precipitate and enrich RNAs with low molecular weight. Next, a 15% polyacrylamide denaturing gel was employed to separate the low-molecular weight RNA. During gel electrophoresis, about 100 μg of total RNA was applied to the gel and two labeled RNA oligonucleotides, approximately 18 and 26 nt in length, were used as size standards. After gel electrophoresis, small RNAs with 18-26 nt were excised from the gel and eluted with 0.4 M NaCl overnight at 4°C. The RNA was dephosphorylated using alkaline phosphatase (New England Biolabs, Beijing China) and recovered by ethanol precipitation. The isolated small RNAs were then sequentially ligated to RNA/DNA chimeric oligonucleotide adapters, reversely transcribed, and amplified by PCR. Finally, Solexa sequencing technology was employed to sequence the small RNAs from pooled peanut samples (BGI, Beijing China).

### Identification of conserved and peanut-specific miRNAs

The raw sequences were processed using PHRED and CROSS MATCH programs as previously reported [21,55]. After removing the vector sequences, trimmed sequences longer than 17 nt were used for further analyses. First, rRNA, tRNA, snRNA, and snoRNA, as well as those containing the polyA tail, were removed from the small RNA sequences and the remaining sequences were compared against rice and *Arabidopsis *ncRNAs deposited in the NCBI Genbank database and Rfam8.0 database. Then, the unique small RNA sequences were used to do a Blastn search against the miRNA database, miRBase 13.0, in order to identify conserved miRNAs in peanuts. Only perfectly matched sequences were considered to be conserved miRNAs. To study potential miRNA precursor sequences, we used the identified peanut mature miRNA sequences to do Blastn searches against peanut ESTs in NCBI. Non-coding ESTs, which met previously described criteria [56], were then considered to be miRNA precursors. Specifically, dominant, mature sequences residing in the stem region of the stem-loop structure and ranging between 20-22 nt with a maximum free-folding energy of -25 kcal mol^-1 ^were considered. A maximum of six unpaired nucleotides between the miRNA and miRNA* was allowed. The distance between the miRNA and miRNA* ranged between 5 and 240-nt. After removing the conserved miRNA sequences, the rest of the small RNA sequences were used to perform Blastn searches against peanut ESTs in order to obtain precursor sequences for novel potential miRNAs. The selected EST sequences were then folded into a secondary structure using an RNA-folding program mFold. If a perfect stem-loop structure was formed, the small RNA sequence was sit at one arm of the stem as well as other criteria were followed, this small RNA was consisted as one novel peanut miRNA.

### miRNA validation

Identified peanut miRNAs were validated using quantitative real time PCR (qRT-PCR) using a well-developed strategy. The Applied Biosystems TaqMan^® ^microRNA Assays (Foster City, CA) were employed to detect and compare the expression levels of miRNAs in peanut leaves. TaqMan-based real time quantification of peanut miRNAs includes two important steps: a reverse transcription reaction and a real time quantitative PCR reaction [52]. In this study, 5 conserved miRNAs (miR156, miR157, miR162, miR172, and miR396) and 4 peanut-specific miRNAs (miRn1, miRn2 and miRn2*, miRn3, and miRn4) were validated using qRT-PCR (Table 3). The primer and probe sequences for the 5 conserved miRNAs were purchased from Applied Biosystems and the sequences of the primers for the 4 peanut-specific miRNAs were obtained from Invitrogen. In the reverse transcription reaction, mature miRNAs were reversely transcribed into cDNAs using a miRNA-specific stem-loop RT primer and a reverse transcriptase enzyme. In the qRT-PCR reaction, the expression levels of the 5 conserved and 4 peanut-specific miRNAs were analyzed using miRNA-specific primers (forward and reverse primers) [52].

**Table 3:** qRT-PCR-validated miRNAs and their sequences

miRNA	Sequence
miR 156	UGACAGAAGAGAGUGAGCAC
miR 157	UUGACAGAAGAUAGAGAGCAC
miR162	UCGAUAAACCUCUGCAUCCAG
miR172	AGAAUCUUGAUGAUGCUGCAU
miR396	UUCCACAGCUUUCUUGAACUG
miRn1	UAGAGGGUCCCCAUGUUCUCA
miRn2	UCACCGUUAAUACAGAAUCCUU
miRn2*	AGGAUUCUGUAUUAACGGUGA
miRn3	AAUGUAGAAAAUGAACGGUAU
miRn4	UGCUGGGUGAUAUUGACAGAAG

The RT-PCR and qRT-PCR reactions, for validating and detecting peanut miRNAs, were followed using the same protocols as our previous report [37,53]. Briefly, miRNA reverse transcription reactions were performed in 200 μL PCR tubes, each containing a total of 20 μL of reaction solution. Each reaction solution contained 1000 ng of total leaf RNAs, 3.33 U/μL MultiScribe reverse transcriptase, 1× reverse transcription buffer, 0.25 mM each of dNTPs, and 0.25 U/μL RNase inhibitor; sterilized RNase-free water was used to adjust the total volume of the reverse transcription reaction to 20 μL. The miRNA reverse transcription reactions were incubated in an Eppendorf Mastercycler (Eppendorf North America, Westbury, NY). The RT-PCR temperature program was adjusted to run for 30 min at 16°C, 30 min at 42°C, 5 min at 85°C, and then 4°C until future use. For each miRNA, three biological replicates were performed. After reverse transcription, the products of each reaction were diluted 10 times to avoid potential primer interference in the following qRT-PCR reaction.

Quantitative real time PCR was performed using the TaqMan^® ^microRNA Assay kit (Foster City, CA) on an Applied Biosystems 7300 Sequence Detection System (Foster City, CA). Each reaction consisted of 3 μL of product from the diluted reverse transcription reaction, 2 μL of 20× TaqMan MicroRNA Assay primers (forward and reverse), 12.5 μL of 2× TaqMan Universal PCR Master Mix, and 7.5 μL of nuclease-free water. The reactions were incubated in a 96-well plate at 95°C for 10 min, followed by 45 cycles of 95°C for 15s and 60°C for 60s. After the reactions were completed, the threshold was manually set and the threshold cycle (C_T_) was automatically recorded. The C_T _is defined as the fractional cycle number at which the fluorescence signal passes the fixed threshold [52]. All reactions were run in two replicates for each sample.

### Target gene prediction

The potential targets of peanut miRNAs were predicted using the psRNATarget program http://bioinfo3.noble.org/psRNATarget/ with default parameters. Newly identified peanut miRNA sequences were used as custom miRNA sequences; *Arachis *transcript/genomic library (EST, GSS, and nucleotide databases) were used as custom plant databases.

All predicted target genes were evaluated by scoring, and the criteria used were as follows: each G:U wobble pairing was assigned 0.5 points, each indel was assigned 2.0 points, and all other non-canonical Watson-Crick pairings were assigned 1.0 points each. The total score for an alignment was calculated based on 20 nt. When the query was longer than 20 nt, scores for all possible consecutive 20 nt subsequences were computed, and the minimum score was considered the total score for the query-subject alignment. Because targets complementary to the miRNA 5' end appear to be critical, mismatches other than G:U wobbles at positions 2-7 at the 5' end were further penalized by 0.5 points in the final score [57]. Sequences were considered to be miRNA targets if the total score was less than 3.0 points.

Once potential target mRNA sequences were obtained, redundant sequences were removed using the 'contig express' feature of the Vector NTI program. Blastx was performed using the target sequence and the NCBI database to predict functions of potential targets.

## Authors' contributions

XW conceived the intellectual design of the project and wrote the manuscript. HX and CZ undertook most of the sequence analysis to identify miRNAs, secondary structures, and prediction of target genes. They also participated in part of the manuscript writing, namely, the method section. TPF and BZ performed the RT-PCR and qRT-PCR experiments and also gave intellectual suggestion for the manuscript writing. YY, YB and AL carried out plant growth, RNA preparation, miRNA library construction. ML and CL completed database searching, data management and processing. All authors read and approved the final version of manuscript.

## Supplementary Material

Additional file 1 Secondary structures of conserved and novel miRNAs in peanuts. Click here for file

Additional file 2 The putative target genes of identified miRNAs. Click here for file
