# Supplementary material for: Deep sequencing identifies novel and conserved microRNAs in peanuts (Arachis hypogaea L.)
Source: BMC Plant Biol. 2010 Jan 5;10:3. doi: 10.1186/1471-2229-10-3 (PMC2826338; doi:10.1186/1471-2229-10-3)
Supplement: Additional file 1 — Secondary structures of conserved and novel miRNAs in peanuts. [file 1471-2229-10-3-S1.RTF]

Additional file 1. Secondary structures of conserved and novel miRNAs in peanuts
I. Secondary structure of peanut conserved miRNAs

(1)ahy-miR156a: UGACAGAAGAGAGUGAGCAC

GA      UAUAA   U           -          A-     G   U   C    A 
  AGAGAU     AUG ugacagaagag agagagcacA  CCCGA AAA GGU AAAG \
  UUUCUA     UAC ACUGUCUUCUC UCUCUCGUGU  GGGUU UUU CCA UUUC A
A-      UUUC-   U           A          GA     G   -   U    U 


(2) ahy-miR157a-5p: UUGACAGAAGAUAGAGAGCAC
ahy-miR157a-3P: gcucucuaagcuucugucauc

-------      ACUAU   U    U  u         a-             A-     U     .-UAUA|  AUA 
       GGAGGC     UGG AGAG UG ugacagaag  uagagagcacAAA  GAUGA AUGCA      GAU   \
       UCUCCG     ACU UCUC Ac acugucuuc  aucucucgUGUUU  UUAUU UACGU      CUG   U
AUCUUUA      -----   -    -  u         ga             CC     U     \ ----^  AUA 


(3) ahy-miR157k: UUGACAGAAGAGAGAGAGCAC

CCUCGA--------------      UAUAA   u           -          A-|    G   U   C    A 
                    AGAGAU     AUG ugacagaagag agagagcacA  CCCGA AAA GGU AAAG \
                    UUUCUA     UAC ACUGUCUUCUC UCUCUCGUGU  GGGUU UUU CCA UUUC A
UUCUAGAUAUGGUAUGUUUA      UUUC-   U           A          GA^    G   -   U    U 


(4) ahy-miR160b-5p: UGCCUGGCUCCCUGAAUGCCA
ahy-miR160b-3p: GCAUGAAGGGAGUCACGCAGG

AAAUA-      UUAUG-|      c         ga            UUA         GAA 
      UUCGAA      UGUCugc uggcucccu  augccaUGUAAG   GUUUGUUAA   \
      AAGUUU      GCggacg acugaggga  uacgGUAUAUUC   CAAACAAUU   A
UAUACA      UACUUA^      c         ag            UG-         AGA 


(5) ahy-miR167f-5p: UGAAGCUGCCAGCAUGAUCUU
ahy-miR167f-3p: AGAUCAUGUGGCAGUUUCACC

GA      A-  -| U  A  U           g        uAA   U    CUCCUAU 
  UCAUGC  CC AC AC AG ugaagcugcca caugaucu   CUU CCCU       \
  AGUACG  GG UG UG Uc acuuugacggu guacuaga   GAG GGGG       G
UA      AA  U^ U  A  c           -        CUA   U    UUGUUUA 


(6) ahy-miR159a: UUUGGAUUGAAGGGAGCUCUA

A------------------            GA       CA        -   U-      AUU     G   AGC      UC  A   U      ----|   UAUA 
                   GUGGAGCUCCUU  AGUCCAA  GAGGGUCU UGG  GGGUAG   GAGCU CUA   UAUGGA  CC CAG ACUACC    CAUA    C
                   Caucucgaggga  uuagguu  UUCCUAGA ACC  UCCAUU   UUCGA GGU   AUACCU  GG GUC UGAUGG    GUAU    C
UAAUCUUUCUCCUACGAUA            ag       uA        C   UU      AC-     G   CCU      UA  C   U      UGAC^   UACC 


(7) ahy-miR394a: UUGGCAUUCUGUCCACCUCC

.-AG   U     -  U    AU      auu   u        .-GAUC|  UC 
    GCA UGUUC CA AGCC  Uuuggc   cug ccaccucc      CGA  C
    UGU AUAAG GU UUGG  AAACCG   GAC GGUGGAGG      GCU  G
\ --   -     U  C    GC      GAU   C        \ ----^  UA 


(8) ahy-miR408-5p: AUGCACUGCCUCUUCCCUGGC
ahy-miR408-3p: CUGGGAACAGGCAGAGCAUGA

-----          U  CAA   A  -     c      a      A  .-GAACUA|    AG   CA 
     GAAGAAGAGA GA   AGA cu gggaa aggcag gcauga UG        UCAAU  ACA  \
     CUUUUUCUUU CU   UCU gg cccuu uccguc cguaCU GC        AGUUA  UGU  U
UCUUC          -  C--   c  u     c      a      C  \ ------^    CU   UU 


(9) ahy-MIR398b: UGUGUUCUCAGGUCGCCCCUG

  .-UAU        A    A             A        U--       UU-  GAA       AUC   U      U   U 
       CUCAGAGG GUGA CCUGAGAACACAA GUAAAUUG   UUCGGAG   UG   UGCCAUA   ACA GCAUAA GCA U
       GAGUuucc cacu ggacucuuguguU CAUUUAAU   AAGCUUU   AC   AUGGUAU   UGU CGUAUU CGU U
  \ ---        c    -             C        UUU       UAU  ---       A--   U      -   A 
 


II. Secondary structure of peanut novel miRNAs

(1)ahy-miRn1£ºUAGAGGGUCCCCAUGUUCUCA

AGGC AA    --   UAUCUU        CA  C  A           C    CUG-   U     .-CUCGUAA     U 
      A  AUUC  UCC      UGAGGACA  GG GA UCCUCUAUUCU UCUU    CAC GCCGC         AUCAC A
      U  UGAG  AGG      acucuugu  cc cu gggagauAAGA AGAA    GUG UGGUG         UAGUG C
  CA-- UN    UC   UUNAAU        ac  -  -           -    ACGA   -     \ -------     U 


(2)ahy-miRn2-5p: UCACCGUUAAUACAGAAUCCUU
ahy-miRn2-3p: AGGAUUCUGUAUUAACGGUGA

ACAAUA--------------|           u       g       CGU        -   UC    C       A       A         AGUUUUU 
                   GAaggauucugua uaacggu gaCAUGA UUUAUCU   UUUUAAAG AUA  UUUG AUUUCAU UGAGAUUUA        A
                   Cuuccuaagacau auugcca cuGUACU AAAUAGA   GAAAUUUU UAU  AAAU UAAAGUA ACUCUAAAU        U
GGGGACUGAUUUAUAUUAA             a^      -       G       AUU        A   UU    U       C         AUAAUGGU 


(3)ahy-miRn3£ºAAUGUAGAAAAUGAACGGUAU

  GGAGGAGAA      ---         C   AU             CUCAUU          UC        -  U     A       AUA 
           UCAAUA   AUGUUAUUU UAC  UUCAUUUUCUAUA      CUCUGCACCU  CUUUUAUG UA GAUAA GGAAAAA   \
           AGUUGU   UACAAUAAA aug  aaguaaaagaugu      GAGAUGUGGA  GAAAAUAC GU UUAUU UCUUUUU   U
  AG-------      AGU         u   gc             aaAAAU          GU        U  U     A       AAA 


(4) ahy-miRn4 :UGCUGGGUGAUAUUGACAGAAG

  .-ug     u      ga        UUCU 
      cuggg gauauu  cagaagUG    \
      GGCCC CUGUGA  GUCUUCAC    U
  \ --     -      A-        CUCC 


(5) ahy-miRn5: CUGACCACUGUGAUCCCGGAA

GCGUAUCUAACCCAAGGAAGU    c    -     ccc   .-aUAACCCA|    U 
                     cuga cacu gugau   gga          AGGGG A
                     GACU GUGG CACUG   CCU          UCCCC C
C--------------------    -    C     CGA   \ --------^    C 


(6) ahy-miRn6: UGACCUUUGGGGAUAUUCGUG

GCUAAGCGACAA           A    AU  A         A     U           A          .-A|  G  AAG    U 
            UAAUUUGAGAG UAAU  GU ACGAGUAUC CCAAA GUCACUCUUUU GAUUUGUCUU   UUA UU   GAGU G
            AUUAAACUCUU AUUA  CG ugcuuauag gguuu caguGAGAAAA UUAAAUAGGA   AAU AA   UUCA C
------------           C    CU  g         g     c           -          \ -^  G  GUA    A 


(7) ahy-miRn7: UCAAUCAAUGACAGCAUUUCA

GA---    UG   A    GCU     -       AGUUCU    .-UUUUUC    AAU 
     UGAA  GCU UCAU   UUGAG UGUUGUG      AAAC        UGUG   A
     acuu  cga agua   aacuU ACGAUAC      UUUG        ACAC   U
UGUUA    ua   c    acu     G       GU----    \ ------    AUA 


(8) ahy-miRn8-5p: UGGUGAUGGUGAAUAUCUUAUC
ahy-miRn8-3p: AAGGGAGACGUUUGAAUUAUC


-    ----------------  GG   .-UCUAUUU     -|   gg    ua    a          UU 
 GACC                UG  UCC         AUAgg ugau  ugaa  ucuu ucUUAUCUUA  U
 UUGG                AC  AGG         UAUcc auua  guuu  agag ggaaUAGAAU  G
A    UUGAAAGCUUAANUUU  AG   \ -------     u^   a-    gc    -          UG 


(9) ahy-miRn9: UGGUGAGUCGUAUACAUACUG

  .-G     UU-      A  U   A- UG     -------       U 
     GGUAU   UGUGAC UU CCG  U  GCUGU       CUUGUUG U
     ucaua   augcug ag ggu  G  UGAUA       GAAUAAU U
  \ g     cau      -  u   AC GU     CUUUGUU       C 


(10) ahy-miRn10-5p: AUACUUGAGAGCCGUUAGAUGA    
     ahy-miRn10-3p: AUCUAACGACUCUCAGAUAUAAU


UC----------------------------------------|         U        a  c       c          UUU    GAUU 
                                          GACUUCACGU GAAGUUGA ua uugagag cguuagauga   GAUU    U
                                          CUGAAGUGCA CUUCAAcu au gacucuc gcaaucuaCU   UUAA    G
GAUUAAGAUAAAAACAUAAUUCCACUUUUGAGUCCACUUCAA^         -        -  a       a          U--    AUCA 


(11) ahy-miRn11: UUAUACCAUCUUGCGAGACUGA

.-CAAA     --|       a   u   gc     ug  CG 
      UGGCU  UGGUuuau cca cuu  gagac  aA  A
      AUCGA  ACCGAGUA GGU GGA  UUUUG  UU  G
\ ----     UU^       -   U   --     GU  UA 
 


(12) ahy-miRn12-5p: UGUUACUAUGGCAUCUGGUAA 
     ahy-miRn12-3p: GCCAGGGCCAUGAAUGCAGAU

        
AAU     C--      -      aau   GAAC-    AAAGG 
   CGAGC   gccagg gccaug   gca     UGUU     \
   GUUCG   uggucu cgguau   ugu     ACAA     A
C--     AAa      a      cau   AACAA    GAAAA
 

(13) ahy-miRn13: CGCAAAUGAUGACAAAUAGA

--------|    AUUAUA  UA  A   CUGG  U       A     ACU---      G     GAAAAAUCCAUG 
        GUUGA      GU  CG UCU    GU CGUUAUU GUGAA      GAGGUA UGUGU            \
        CGACU      CA  GC aga    ca guaguaa cgcUU      CUUUAU ACGUA            U
GAAUCUCA^    A-----  GG  -   uaaa  -       a     AUCAUU      G     AACGUUCGAAUU 


(14) ahy-miRn14-5p: UUAAUUUCUGAGUUUGUCAUC
ahy-miRn14-3p: UUGAUAAGAUAGAAAUUGUAU 


UAUUGUCUAAUU         -    ---|        ag       ucU     UCUGUUGAU    UUCUAAAU 
            GUAAU--GG AUUU   uaauuucug  uuuguca   GAACU         UGUG        \
            CGUUG  CC UAAA   guuaaagau  gaauagu   UUUGG         ACAU        U
U-----------     \   A    UAu^        a-       uG-     UUGUUGUGU    UGUAGUCU 


Red colored letter: mature miRNA sequence
Blue colored letter: miRNA* sequence
