# Supplementary material for: Deep sequencing identifies novel and conserved microRNAs in peanuts (Arachis hypogaea L.)
Source: BMC Plant Biol. 2010 Jan 5;10:3. doi: 10.1186/1471-2229-10-3 (PMC2826338; doi:10.1186/1471-2229-10-3)
Supplement: Additional file 2 — The putative target genes of identified miRNAs. [file 1471-2229-10-3-S2.DOC]

Additional file 2. The putative target genes of identified miRNAs

| **miRNA family** | **Target EST** | **Score** | **Annotation** | **E-value** |
| --- | --- | --- | --- | --- |
| ahy-MIR156 | ES767441 | 1 | Squamosa promoter-binding protein, putative [*Ricinus communis*] | 6e-29 |
| ahy-MIR164 | GO265825 | 1 | NAC domain protein NAC1 [*Phaseolus vulgaris*] | 1e-111 |
|  | ES712265 | 1.5 | NAC domain protein [*Glycine max*] | 4e-103 |
|  | EH042413 | 1.5 | NAM (no apical meristem)-like protein [*Arabidopsis thaliana*] | 7e-31 |
| ahy-MIR167 | ES717439 | 1 | AP-2 complex subunit alpha [*Schizosaccharomyces japonicus yFS275*] | 9.7 |
|  | GO341071 | 3 | Similar to auxin response factor 8 [*Vitis vinifera*] | 6e-81 |
| ahy-MIR169 | ES718171 | 2 | Nuclear transcription factor Y subunit A-3, putative [*Ricinus communis*] | 2e-18 |
|  | EE124598 | 2.5 | Nuclear transcription factor Y subunit A-3, putative [*Ricinus communis*] | 4e-37 |
| ahy-MIR171 | GO324473 | 0.5 | GRAS family transcription factor [*Populus trichocarpa*] | 4e-16 |
| ahy-MIR172 | EH042284 | 0.5 | 窗体顶端  Protein AINTEGUMENTA, putative [*Ricinus communis*] 窗体底端  窗体顶端 | 2e-26窗体底端 |
| ahy-MIR390 | GO343040 | 2.5 | Protein kinase [*Glycine max*] | 8e-113 |
|  | ES723914 | 2 | No hit found |  |
| ahy-MIR393 | ES718695 | 1 | Auxin signaling F-box 3 [*Arabidopsis thaliana*]; f-box family protein [*Populus trichocarpa*] | 2e-90 |
|  | ES758140 | 1.5 | TRANSPORT INHIBITOR RESPONSE 1 protein, putative [*Ricinus communis*] | 1e-40 |
| ahy-MIR396 | ES721690 | 1 | Growth regulating factor 5 [*Arabidopsis thaliana*] | 8e-40 |
|  | ES724458 |  | Growth-regulating factor 5 [*Oryza sativa* (*japonica cultivar-group*)] | 4e-36 |
| ahy-MIR397 | GO257848 | 1 | Laccase 110c [*Populus trichocarpa*] | 4e-85 |
|  | EH044190 | 3 | Resveratrol synthase [*Arachis hypogaea*] | 6e-119 |
| ahy-MIR398 | EE125712 | 3 | Serine hydroxymethyltransferase 2 [*Glycine max*] | 5e-80 |
| ahy-MIR408 | EH046053 | 2 | Basic blue protein, putative [*Ricinus communis*] | 1e-42 |
| ahy-MIR528 | ES755121 | 2.5 | Unknown [*Glycine max*] | 2e-45 |
| ahy-miRn3 | ES721338 | 2.5 | Similar to Pyrophosphate-fructose 6-phosphate 1-phosphotransferase subunit alpha [*Vitis vinifera*] | 2e-62 |
| ahy-miRn4 | EG028791 | 2.5 | Unknown [*Glycine max*] | 5e-75 |
| ahy-miRn8 | GO335533 | 2.5 | Heme oxygenase 2 [*Arabidopsis thaliana*] | 2e-56 |
|  | GF100622 | 1.5 | Disease resistance response protein, putative [*Ricinus communis*] | 2e-23 |
| ahy-miRn9 | GO338394 | 0 | ATP-dependent Clp protease proteolytic subunit [*Lotus japonicus*] | 4e-40 |
|  | ES761917 |  | unnamed protein product [*Vitis vinifera*] | 5e-17 |
|  | ES760701 |  | Ribosomal protein S12 [*Medicago truncatula*] | 3e-11 |
| ahy-miRn10 | EL966894 | 1.5 | Full length desiccation protectant protein Lea14 homolog [*Glycine max*] | 3e-35 |
| ahy-miRn12 | GO343422 | 2.5 | Exonuclease, putative [*Ricinus communis*] | 1e-66 |
| ahy-miRn14 | EG029660 | 0 | Epoxide hydrolase [*Glycine max*] | 5e-108 |
